# Investigation of corrosion and water absorption of biomass natural coir fiber/hBN reinforced epoxy hybrid composites using different optimisation approaches

**DOI:** 10.1038/s41598-025-87673-6

**Published:** 2025-01-27

**Authors:** Ramraji Kirubakaran, Shenbaga Velu Pitchumani, Sanju Tom, R. Ashwin Nagaraj, P. Salin, Harish Kumar Natchimuthu, Venkatachalam Gopalan, G. Vinayagamurthy

**Affiliations:** 1 School of Mechanical Engineering, Vellore Institute of Technology, Chennai, 600127 Tamilnadu India; 2 Centre for Advanced Materials and Innovative Technologies, Vellore Institute of Technology, Chennai, 600127 Tamilnadu India

**Keywords:** Coir fiber and hBN filler, Box-Behnken Design, Polymer composite, Metaheuristic algorithms, Corrosion behaviour, Water absorption behaviours, Structural materials, Mechanical engineering, Statistics

## Abstract

Agricultural waste or agro-waste, including natural fibers and particles from various crop parts, is increasingly recognized as a significant contributor to environmental issues. However, from a circular economy perspective, these materials present an opportunity to be repurposed into new, eco-friendly products. The present study, specifically focuses on understanding the effect of different factors, such as the particulate loading and the size (coir and hBN − 1 to 5 wt%; Coir Powder size (100–200 μm) of the particles on composite’s corrosion rates and water absorption properties. These hybrid particulate composites (HPC) are fabricated using the hand layup process. The study uses a Box-Behnken Design (BBD-L15), a statistical experimental design tool that facilitates the effective investigation of many input parameters and their interactions, to comprehensively investigate these impacts. In addition, the study utilizes four metaheuristic algorithms—the Dragonfly Algorithm (DFO), the Salp Swarm Algorithm (SSA), Teaching Learning Optimization (TLO) and Particle Swarm Optimization (PSO)—alongside regression equations to predict the optimal characteristics of the composite material. To determine the best-performing algorithm, a comparison is made using Deng’s method. The findings indicate that the composite with a higher weight% of hBN particulates exhibits reduced water absorption and corrosion rates. A larger Deng’s Value often indicates better performance. Based on its higher Deng’s Value, the SSO algorithm outperforms other algorithms in minimizing both corrosion resistance (CR) and water absorption (WA). The Deng’s Value for SSO reached a maximum of 0.68, while the other algorithms show comparable but lower performance.

## Introduction

The materials, known as Fiber Reinforced Polymer (FRP) composites, composed of a polymer matrix reinforced with fibers, have drawn attention lately because of their improved mechanical qualities, damping and durability. These composites show excellent specific properties like high strength-to-weight ratios, good thermal stability and biodegradability when they are reinforced with natural fibers (NFs) like jute, hemp, flax, or banana fibers, or natural particles (NPs) like rice husk, coir, coconut shell, or seaweed^1–3^. They also offer sustainable and environmental friendly alternatives to synthetic reinforcements. Because of these qualities, composites reinforced with natural fiber or particles can be used in a variety of sectors and have a broad range of applications^4^. However, natural fillers have water absorption tendency (hydrophilic behaviour) due to that hemi cellulose, lignin content besides suitable surface modification to reduce the hydrophilic behaviour^5,6^.

On the other hand, ceramic particulate (like boron nitride-BN, titanium carbide, silicon carbide-SiC, aluminum oxide-Al_2_O_3_, Silicon nitride -Si_3_N_4_ and aluminium nitride-AlN) with polymer matrix enhances the mechanical, tribology, thermal and corrosion properties of the composites^7–10^. The typical features of hexagonal boron nitride (h-BN), a two-dimensional (2D) material that bears similarities to graphene, has attention in the field of metal corrosion protection. Because of its layered structure, it has exceptional barrier qualities that successfully obstruct the flow of liquids and gases. The material’s exceptional resistance to corrosion is mostly due to its exceptional impermeability, which keeps corrosive substances like water, oxygen and other chemicals from penetrating the metal surface. Because of these qualities, h-BN is a good option for applications that need strong protective coatings in challenging settings. Since hexagonal boron nitride (h-BN) is a superior electrical insulator compared to conductive materials such as graphene, it can efficiently block galvanic corrosion. In settings where metal connections are exposed to moisture or electrolytes, h-BN is especially useful because it stops the electrochemical reactions that cause corrosion by obstructing the flow of electrons between dissimilar metals. Because of its nonpolar and layered structure, hBN has a naturally hydrophobic surface that minimises its affinity for molecules of water. The entry of water into the composite material is reduced when hBN particles are integrated into a polymer matrix because they produce a barrier effect. This hydrophobic nature of hBN ceramic particleplays a major role in decreased water absorption^11–13^. Furthermore, h-BN has strong mechanical properties, anti-friction vibration characteristics and excellent thermal stability, making it a material with potential uses in lubrication and wear resistance^12,14^.

Epoxy polymer has been used extensively as a protective coating to stop metal corrosion because of its exceptional electrical insulating qualities, robust adherence to a variety of substrates, superior processability and chemical resistance. Furthermore, the epoxy matrix’s resistance to corrosion has been greatly improved by the use of micro/nano ceramic fillers^15^. The anti-corrosion characteristics of epoxy (EP) coatings modified with nano-sized SiO₂ particles were examined in the work by Li et al.^16^. Similar to this, Mostafaei et al.^17^ investigated that the polyaniline coatings improved with ZnO nanoparticles exhibited anti-corrosion properties. According to the results of both studies, nanocomposite coatings have two possible uses: either they can act as physical barriers that keep the substrate away from corrosive environments, or they can act as inhibitory pigments that increase the metal substrate’s corrosion potential and thereby reduce the rate of corrosion. The tendency of nanoparticles to agglomerate due to their large surface area, however, is a common difficulty observed in these investigations. This makes it more difficult to achieve a uniform dispersion and often restricts the maximum content of nanoparticles in the coating to less than 1.0 wt%. Shaikh et al.^18^ studied the effects of epoxy nanocomposites, hBN and cetyltrimethylammonium bromide on adhesion, corrosion and tribological performance. The best results were obtained with a coating containing 3 wt% hBN, applied at 3 mV for 30 min, which showed the lowest friction coefficient (~ 0.42) and improved resistance to corrosion and wear. Xu et al.^14^ found that adding graphite, SiC and PTFE to EP-based composite coatings significantly improved their anti-wear and anti-corrosion properties.

Statistical experimental design techniques are widely used in both academic and industrial settings to examine processes, optimize performance and regulate input variables in order to produce superior outcomes. Response Surface Methodology (RSM), one of the many experimental design techniques, has been extensively used in several domains to enhance composite production procedures^19,20^. RSM, a collection of mathematical and statistical methods, is helpful in modeling and analyzing situations in which multiple variables affect an interest response. Certain RSM designs, like the Box-Behnken Design (BBD) and the Central Composite Design (CCD), are especially useful for examining the connections between various process factors and those links affect performance outcomes. Numerous researches have used these techniques to enhance the manufacturing parameters of composites comprised of various materials, offering insightful information about enhancing material attributes and process effectiveness. Specifically, multi-objective optimization, a potent technique, enables the simultaneous optimization of many objectives, such as optimizing mechanical strength and reducing weight^21–23^. Furthermore, the differential evolution optimization of metaheuristic algorithms plays a crucial role in generating optimized designs for various structural elements^24–26^.

Researchers have employed various techniques for optimizing processes with single and multi-response objectives, including Taguchi’s approach, neural networks, grey theory^27,28^, moth-flame optimisation (MFO) algorithm^29^, Marine Predators Algorithm (MPA)^30^, hunger game search (HGS) algorithm^31^, genetic algorithms^32,33^, arithmetic optimization algorithm^34^, INFO algorithm^35^, prairie dog optimization algorithm (PDOA)^36^, hummingbird-simulated annealing algorithm^37^, Runge Kutta optimization (RUN) algorithm^38^, Biogeography-Based Optimization (BBO)^39^, Particle Swarm Optimization (PSO)^40^, Salp Swarm Optimization (SSO) and Teaching-Learning Based Optimization (TLO)^41,42^, among others. Krink et al.^43^ improved the Particle Swarm Optimization (PSO) algorithm by adding a new particle model called SEPSO (Self-Enhancing Particle Swarm Optimization) in order to overcome the problem of premature convergence in repeated optimization processes. Using a number of benchmark problems that have been well examined in the literature, this sophisticated model was contrasted with the conventional PSO. The comparison showed that SEPSO works to prevent early convergence and produce more dependable optimization outcomes. This enhancement is especially useful for applications involving composite material optimization, where it’s critical to strike the ideal balance among the various performance attributes.

Cellulose-based coir fibers, easily extracted from abundant biomass, are renewable, environmental friendly and widely used in various industries. However, the literature reveals limited research on epoxy hybrid polymer composites reinforced with coir fiber and ceramic particles. Although the corrosion rates and water absorption properties of composites reinforced boron nitride and other natural fibers have been previously studied, the combined effect of using both has rarely been studied. No optimization studies have been conducted on the corrosive properties of natural coir and hBN particulates. The objective of this work is to realize and characterize hybrid polymer samples (HPS) made of coir and hexagonal boron nitride (hBN) and to study their corrosion rates and water absorption behaviours. This study investigates the effects of two different types of particles: natural coir fiber and ceramic hBN. The coir fiber is pulverized and sieved to obtain three different particle sizes. Three factors are considered in this study: coir particle size, coir particulate loading and hBN particulate loading. RSM analysis is performed to create an L15 Box-Behnken Design. Moreover, the tested corrosion and water absorption behaviours are predicted and validated using hybrid different deep neural network-based algorithms BBO, PSO, SSO and TSO. The goal is to enhance the accuracy and efficiency of forecasting CR and WA, which are crucial for the performance of composite materials, by evaluating the effectiveness of these different algorithms.

## Materials and methods

### Material and fabrication process of coir fiber/hBN/epoxy composite

**Fig. 1:**
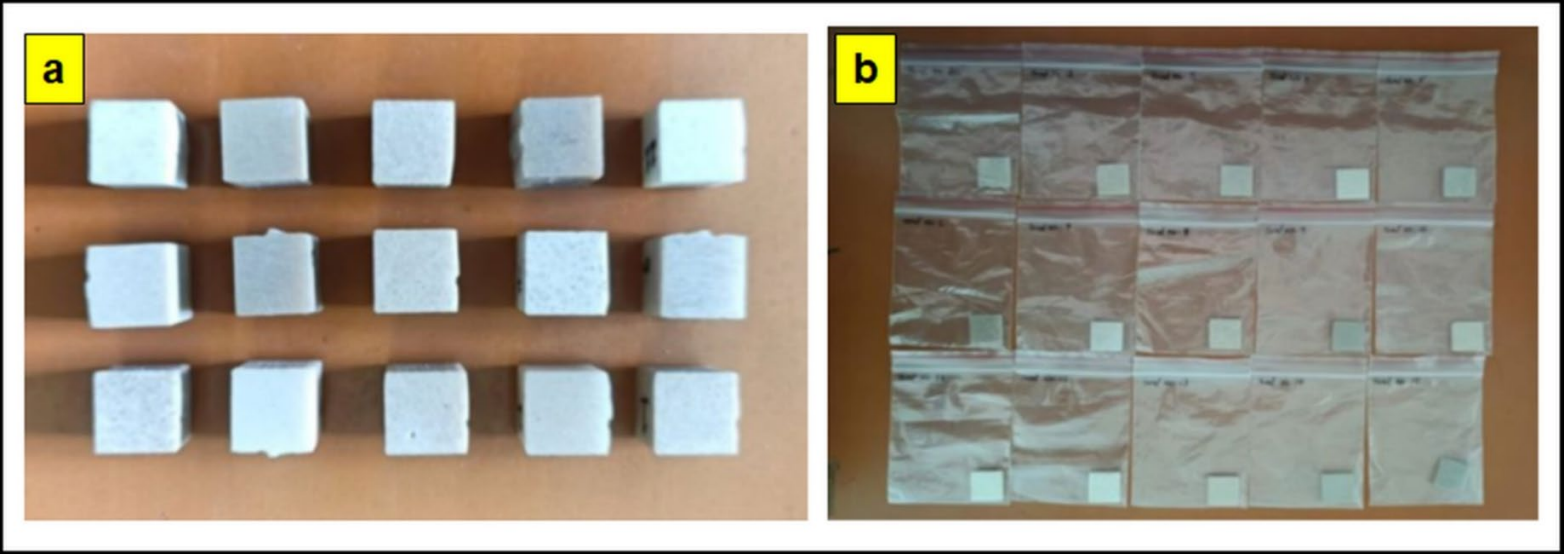
(a) Particulate polymer composite samples for corrosion (b) water absorption test.

This study investigates the polymer matrix composites made from coir fiber/hBN/Epoxy. The study also analyses their responses to corrosion and water absorption properties. CW, CS, BW, CR and WA represent of coir powder, coir powder size, boron nitride, corrosion rates and water absorption respectively. According to the Box Behnken design, coir fiber and hBN powder reinforced with epoxy to make Particulate polymer composite (PPC-15 samples) fabricated through hand layup process. Figure 1 (a, b) shows the prepared particulate polymer composite samples for corrosion and water absorption tests. Table 1 shows the experimental design - parameters with proportion weight% of coir fiber/hBN and its responses.

**Table 1:** Various parameter levels and particle size of coir fiber, wt% of coir fiber particulate and hBN particulate.

Factors	Input Parameters		Levels	
		-1	0	1
CW	Coir fiber wt%	1	3	5
CS	Coir particle size (µm)	75	150	225
BW	HBN wt%	1	3	5

### Corrosion test

**Fig. 2:**
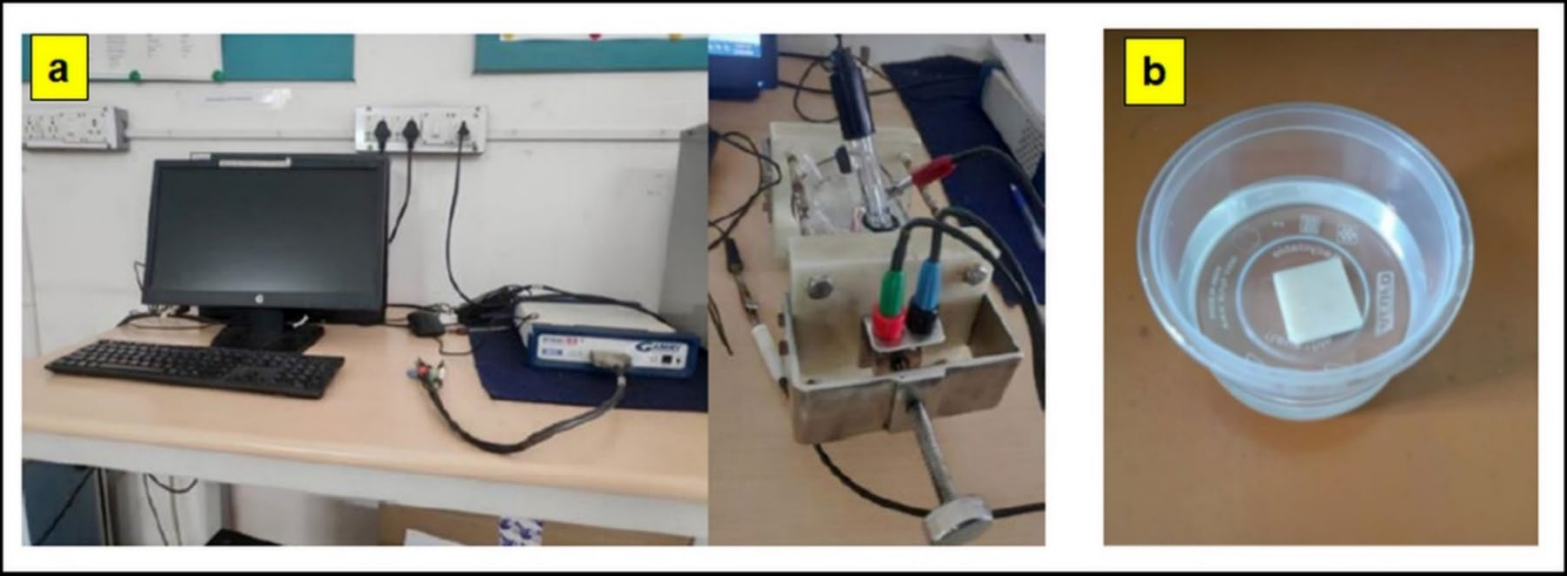
(a) Gamry Potentiostat setup for corrosion test (b) Water absorption test setup.

The corrosion rate is calculated using Gamry potentiostat (Gamry Instruments, Reference 600 Potentiostat/ galvanostat/ 2RA) in 3.5 wt% of NaCl solution. The sample size is 10 × 10 × 10 mm^3^. The sample is connected to the working electrode, the calomel electrode to the feed electrode and the graphite to the counter electrode. To obtain the open circuit potential (OCP), the samples are held for 30 min to stabilise the corrosion potentials prior to the potentiostat test. The potentiodynamic polarisation is carried out at a measurement rate of 1 mV/s. Corrosion potential (*E*corr) and corrosion current density (*I*corr) are obtained using Gamry Echem Analyst software. From the Tafel curves, the corrosion rates are derived. Figure 2 (a) shows the Gamry Potentiostat experimental setup for corrosion test.

### Water absorption

The sample size for water absorption test is 20 × 20 × 3 mm^3^. First step is to remove complete moisture content from composite; to remove moisture the sample is kept in oven at 100 °C for 24 h. The weight of the sample is measured, then the sample is immersed in distilled water for 24 h. After taking out from distilled water, the weight of the sample is measured again and calculated the water absorption percentage. Figure 2 (b) shows the water absorption test.

4.1 $$\:Water\:absorption\:tendency=\frac{{M}_{f}-{M}_{i}}{{M}_{i}}*100$$

where, M_i_ and Mf the starting and finishing weight, both before and after the water immersion.

### Optimization process

This investigation makes use of four distinct algorithms, namely BBO, PSO, SSO and TLO based Optimization and the Parameters of Optimization Algorithms is given in Table 2. For all the algorithms, parameters of population size and number of iterations have fixed values of 50 and 100 respectively. There is a general assumption, followed by the majority of current PSO variants that PSO algorithms perform best with the population size set between 20 and 50 particles. This does not mean, of course, that the impact of the swarm size on PSO performance has never been considered; it was addressed a few times in early studies^44,45^. Then the mutation probability = 0.01; the stopping gap (after which a tournament selection against 20% of the population size is applied); for more details the source paper^46^ can be referred. A common phenomenon is that the control parameters usually are set to different values in solving different optimization problems with different characteristics. Taking the variants of PSO as examples, in^47^, inertia weight fluctuates between 0.1 and 1.0, the local acceleration coefficient is 2.0, global acceleration coefficient is 2.0;^48^.

**Table 2:** Parameters of optimization algorithms.

Algorithms	Parameter	Value
O_BBO, PSO, SSO and TLO	Population Size	50
	No. of Iteration / Epoch	100
O-BBO	Mutation Probability	0.01
	Number of Elites	2
PSO	Local Coefficient	2
	Global Coefficient	2
	Minimum Inertia Weight	0.4
	Maximum Inertia Weight	0.9

#### BBO - biogeography-based optimization

**Fig. 3:**
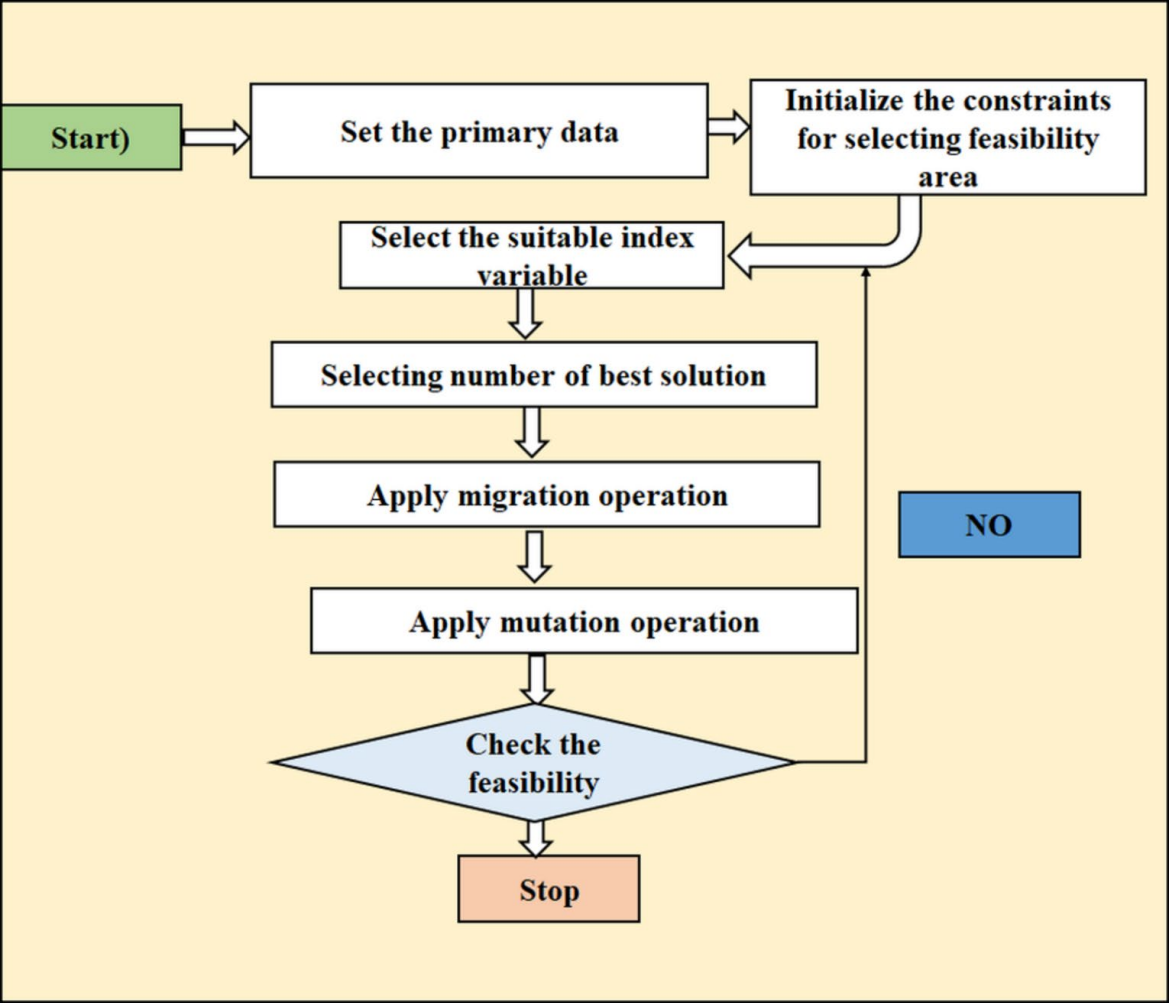
BBO flow diagram image.

The optimisation technique, known as Biogeography-Based Optimisation (BBO), draws inspiration from the field of biogeography, which is concerned with the distribution of various species across various ecosystems. BBO uses the ideas of habitat suitability and species movement to address optimisation challenges. Figure 3 shows the BBO flow diagram image^39^. Every possible solution in the search space is denoted by a “habitat,” and a habitat suitability index (HSI) is used to assess the quality of each habitat. Similar to an environment supporting a bigger number of species, a higher HSI denotes a superior solution^43^. Each algorithm (BBO, TLBO, SSO, etc.) is performed for 100 independent optimization runs, following standard practices in the literature to ensure statistical reliability and account for the stochastic nature of meta-heuristic algorithms. The performance is evaluated using metrics such as the mean best fitness value, standard deviation (for stability), median fitness value (for central tendency) and best/worst fitness values (to capture extremes), along with convergence analysis to track the mean fitness over iterations. In terms of statistical performance, among the algorithms Dragonfly Algorithm (DFO), Salp Swarm Algorithm (SSA), Teaching Learning Optimization (TLO) and Particle Swarm Optimization (PSO), PSO generally demonstrates strong performance across a wide range of optimization problems, often considered a reliable choice due to its balance between exploration and exploitation; However, depending on the specific problem, SSA can sometimes outperform PSO, particularly when dealing with complex, high-dimensional search spaces, while DFO can also show good results with its unique swarm dynamics and TLO can be effective in problems where knowledge transfer is crucial. Overall, these algorithms exhibit varying strengths, with DFO and SSA emerging as notable contenders in the realm of optimization techniques^49^.

The algorithm models to animals move across different habitats. By migrating to different habitats, environments with high HSI typically transfer their “features” (solution qualities), enhancing the quality of habitats with lower HSI. Through time, low-quality ideas get better thanks to this sharing process. Mutation Process: A mutation process is also incorporated in order to preserve population variety and prevent local optima. By introducing random variations into the solutions, this approach can aid in the exploration of novel search space regions. While the mutation guarantees the search for new solutions, the migration assists in the best solution’s exploitation by exchanging advantageous qualities. By striking a balance between exploration and exploitation, the algorithm may find optimal solutions quickly and effectively.

#### PSO – particle swarm optimization

**Fig. 4:**
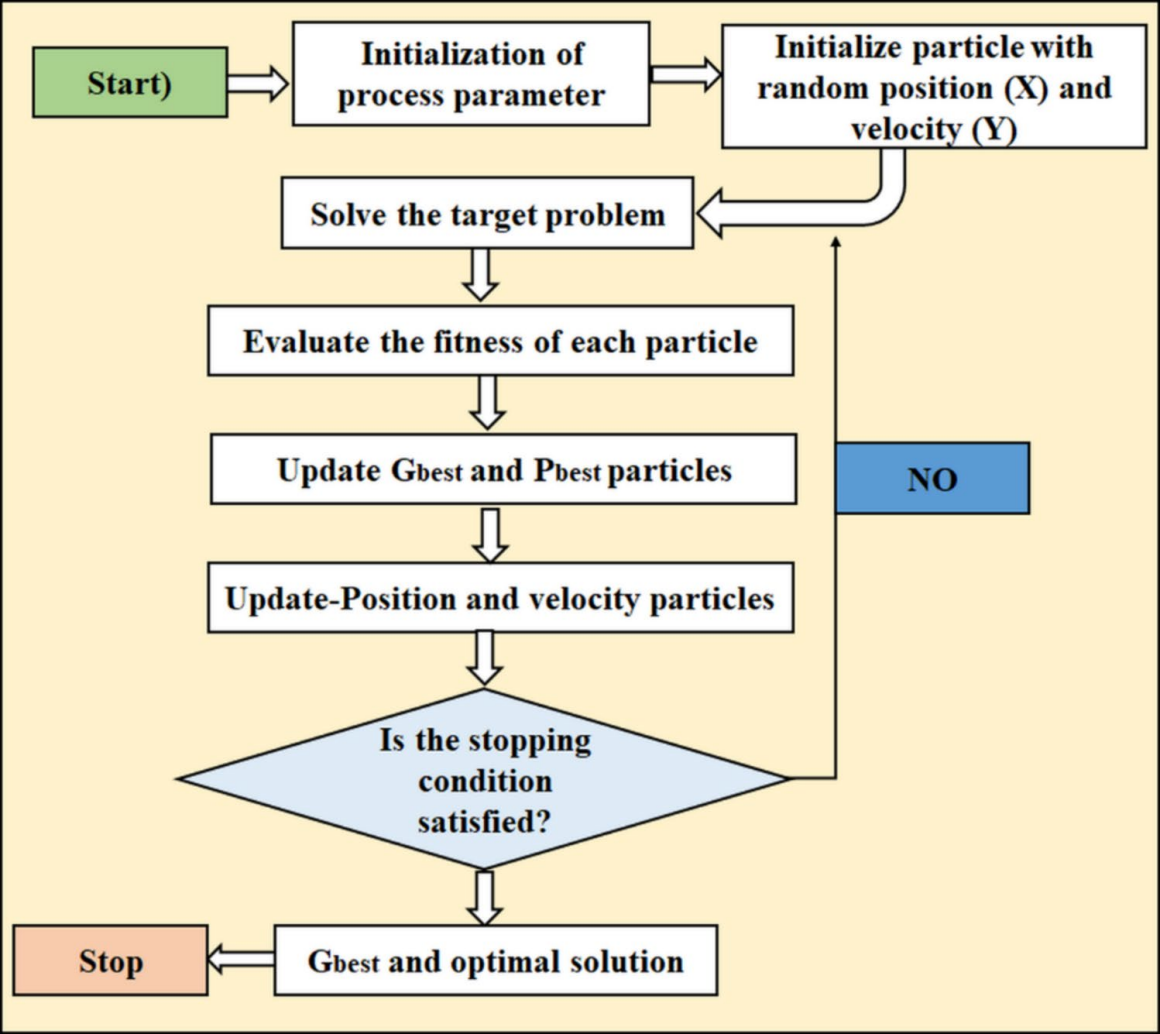
PSO flow diagram image.

The empirical optimisation method, known as Particle Swarm Optimisation (PSO), is motivated by the collective behaviours exhibited by biological groups, such as fish schools or flocks of birds. Figure 4 shows the PSO flow diagram image^40^. The iterative adjustment of particle placements within a search space is the strategy used by this population-based metaheuristic approach to optimise a problem. Every particle moves across the search space, adjusting its position and speed according to its own experiences as well as those of the group’s top performers. The swarm is able to converge towards the best solution thanks to the particles’ collaboration. The two primary factors that control particle motions are cognitive and social. A particle is guided by its cognitive component towards its most well-known position and by its social component towards the optimal position determined by the swarm. PSO is flexible for resolving optimisation issues involving both discrete and continuous variables because it does not rely on gradient information^42,50,51^.

#### Salp swarm optimization

**Fig. 5:**
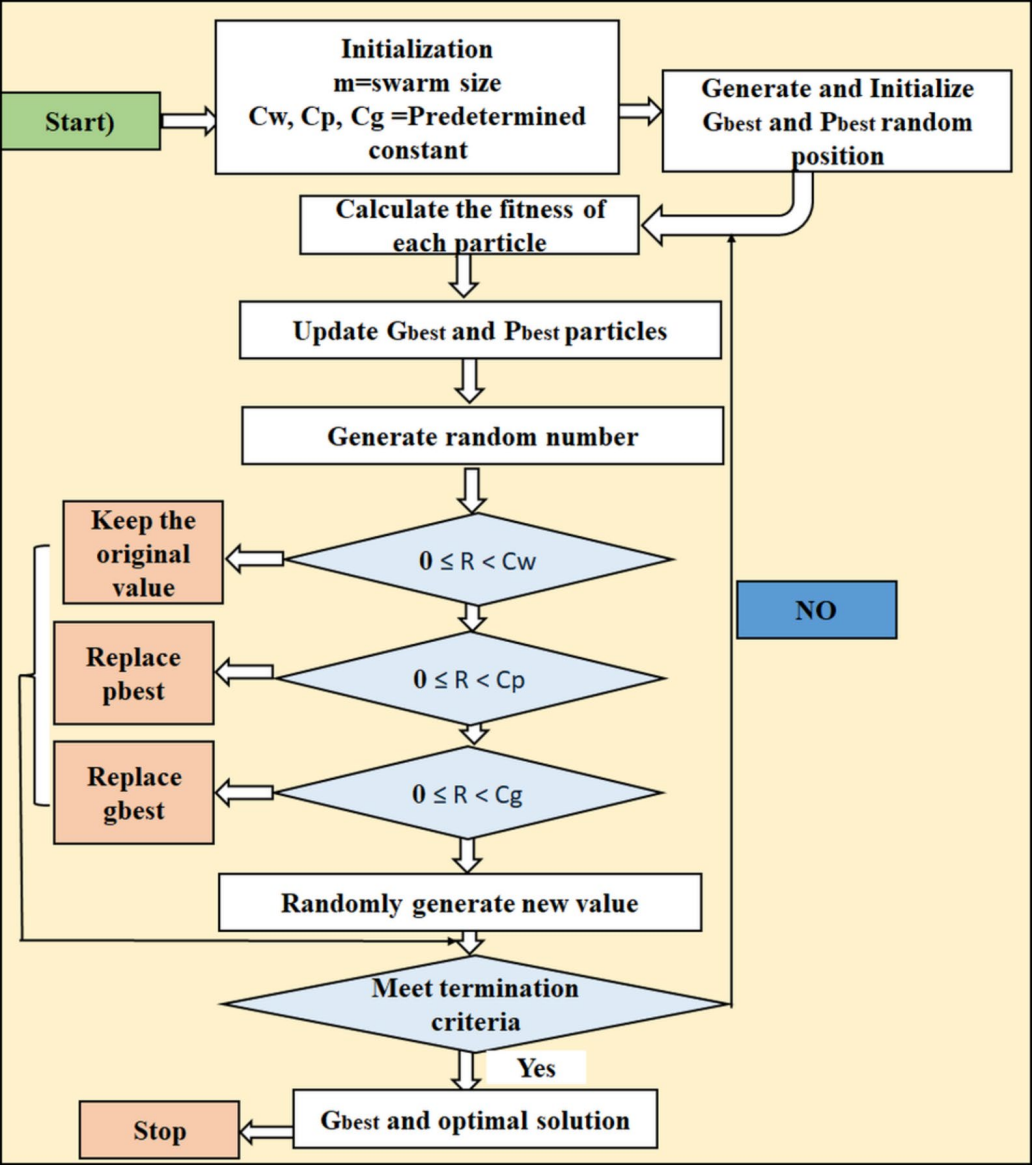
SSO flow diagram image.

The SSO is a population-based optimisation technique developed by Mirjalili^36^ to mimic the chained navigation and hunting of salps in oceans. Figure 5 shows an illustration of the salp chain flow diagram. Theory does not succinctly and clearly address the problem. The salp swarm’s leader controls the population’s movement and each individual follows in his wake. The answer that best aligns with the swarm’s search goal and most effectively responds to the query within the search space has been found by SSO^35–38^.

#### TLO – teaching learning based optimization

**Fig. 6:**
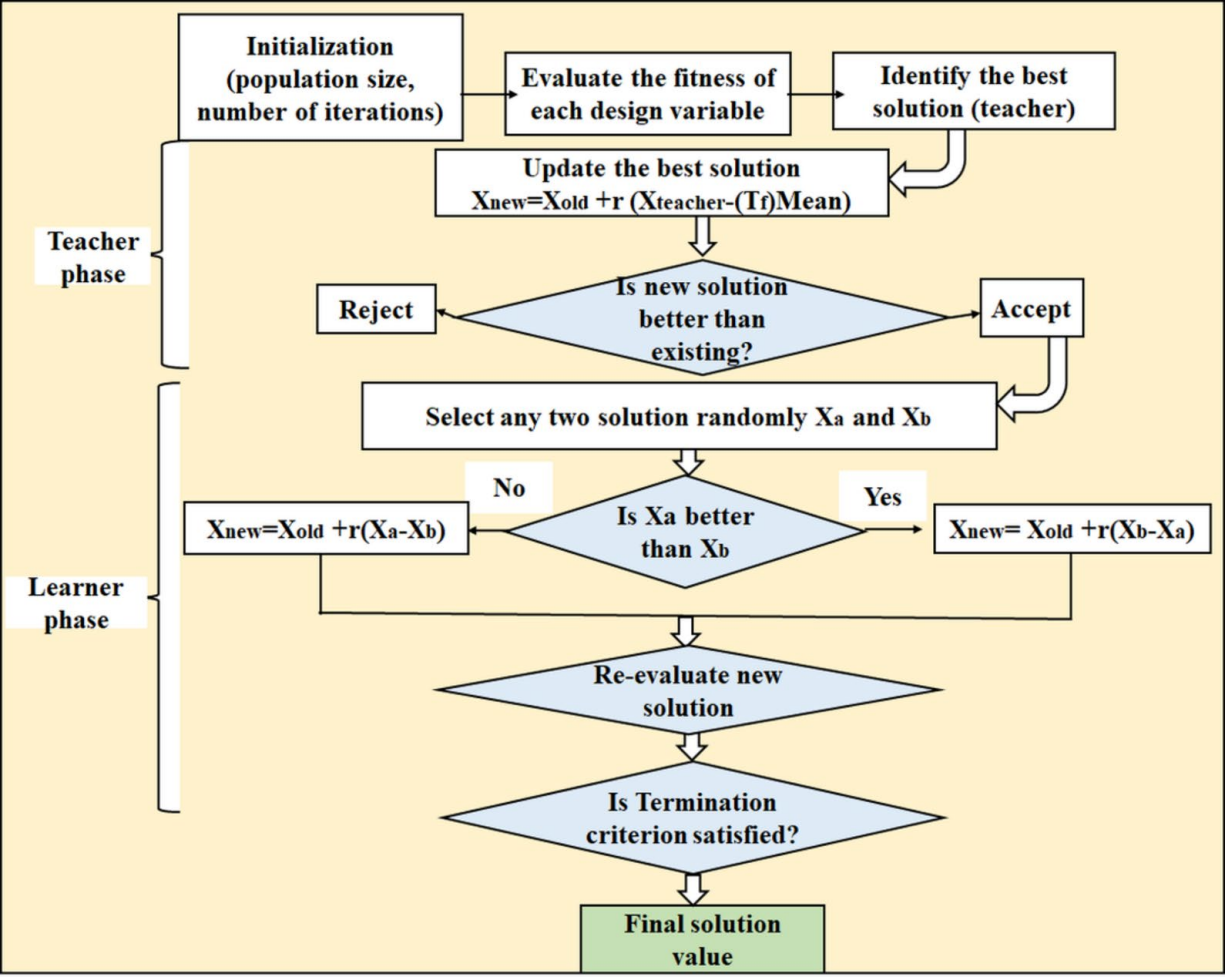
TSO flow diagram image.

The novel optimisation technique, known as Teaching-Learning Based Optimisation (TLO), is motivated by the dynamics of the classroom, specifically the interactions between the teachers and the students. The algorithm works in two main phases: the Learning Phase, where students collaborate to refine their solutions based on peer performance, simulating collaborative learning and the Teaching Phase, where the best-performing solution (the teacher) influences and elevates the performance of the other solutions (students) by adjusting their positions closer to the teacher’s solution. Figure 6 shows the TLO flow diagram image^38^. In contrast to conventional optimisation techniques that mainly depend on subjective assessment, including Taguchi technique-based approaches, TLO is thought to be more efficient and objective. It uses natural processes as guidance for its operations and uses a population-based approach to find the best solutions. When compared to previous evolutionary algorithms, such as artificial bee colonies, Rao et al.^52^ creation, TLO has demonstrated notable advantages, especially when it comes to population size, generation count and computational efficiency.

## Results and discussions

### Experimental results and ANOVA analysis for corrosion rate and water absorption

Table 3 displays the experimental setup, the weight percentages of hexagonal boron nitride (hBN) and coir fiber and the accompanying corrosion rate (CR) and water absorption (WA) responses. The information shows that these characteristics vary significantly between samples. With a value of 233.7 mpy, samples 3, 8 and 13 have the highest corrosion rates, indicating that their composition may be more prone to deterioration in corrosive settings. Sample 14 has the lowest corrosion rate, measuring 0.01 × 10^− 3^ mpy, indicating its better resistance to corrosion. This may be due to an ideal proportion of hBN content and coir fiber, which increases the material’s longevity.

In terms of water absorption, which is an indicator of the material’s hydrophilicity, sample 3 records the maximum water absorption capacity, reaching 1.481%. This shows that the sample may have a less dense microstructure or a higher concentration of hydrophilic components, which could explain its unusually high affinity for water. Sample 11, on the other hand, has the lowest water absorption capacity (0.47%), suggesting that its composition or more compact structure efficiently reduces the amount of water absorbed. The variation in CR and WA between the samples highlights that the weight percentages of coir fiber and hBN affect the composite material’s overall performance and suitability for particular applications, particularly in settings where corrosion protection and moisture resistance are crucial.

**Table 3:** Experimental design - parameters with proportion weight% of coir fiber/hBN and its responses.

E.NO.	CW	CS	BW	CR (x 10^− 3^) (mpy)	WA (%)
1	5	150	5	0.96	1.04
2	1	150	1	0.72	0.59
3	3	150	3	2.34	1.48
4	1	75	3	0.03	0.59
5	5	225	3	0.06	1.32
6	3	225	1	0.30	1.22
7	3	225	5	1.75	1.14
8	3	150	3	2.34	1.28
9	3	75	1	0.85	0.72
10	3	75	5	0.70	0.83
11	1	150	5	0.90	0.47
12	5	150	1	0.15	0.68
13	3	150	3	2.34	1.27
14	5	75	3	0.01	0.94
15	1	225	3	0.02	0.85

The statistical analyses of RSM and ANOVA (Tables 4 and 5) examine the test condition design as well as the output responses (CR and WA). The use of ANOVA to determine the critical factors and the optimal component design for reducing particulate polymer composite corrosion rate (CR) and water absorption (WA) behaviours. The investigation focuses on elements such as hexagonal boron nitride (hBN) particles, weight loading of coir and coir particle size. Major factors are those that contribute over 90% of the cumulative effect to the variable. Based on p-values, ANOVA is utilised to determine whether there is a significant interaction between these components and the CR / WA. The findings, displayed in Tables 4 and 5, describe in detail of weight loading and particle size of the hBN and coir affect on CR and WA. The aim is to comprehend the intricate relationships between various factors in the particulate polymer composite. For composites combining ceramic and natural fillers, interactions like CW*CS (coir weight and coir size), CW*BW (coir weight and BN weight) and CS*BW (coir size and BN weight) are significant factors in the quadratic model of CR and WA properties. The quadratic models created for the CR and WA of coir/hBN composites are statistically significant for various coir sizes (75, 150 and 225 μm) and weight loadings (1%, 3% and 5%), as indicated by the F-value of 81.25 for CR and F-value of 18.85 for WA (p-value < 0.05)^53^.

**Table 4:** Analysis of variance (ANOVA) results for corrosion rate.

Source	DF	Adj SS	Adj MS	F-Value	*P*-Value
Regression	9	0.000011	0.000001	81.25	0.000
CW	1	0.000003	0.000003	195.45	0.000
CS	1	0.000002	0.000002	157.51	0.000
BW	1	0.000000	0.000000	3.16	0.136
CW*CW	1	0.000006	0.000006	396.11	0.000
CS*CS	1	0.000004	0.000004	269.85	0.000
BW*BW	1	0.000001	0.000001	38.85	0.002
CW*CS	1	0.000000	0.000000	0.06	0.820
CW*BW	1	0.000000	0.000000	6.58	0.050
CS*BW	1	0.000001	0.000001	42.97	0.001
Error	5	0.000000	0.000000		
Lack-of-Fit	3	0.000000	0.000000	*	*
Pure Error	2	0.000000	0.000000		
Total	14	0.000011			
Model summary	S = 0.00012	R^2^ = 99.32%	R^2^(adj) = 98.10%	R-sq(pred) = 89.13%	

**Table 5:** Analysis of variance (ANOVA) results for water absorption.

Source	DF	Adj SS	Adj MS	F-Value	*P*-value
Regression	9	1.35105	0.150117	18.85	0.002
CW	1	0.24502	0.245024	30.77	0.003
CS	1	0.04389	0.043895	5.51	0.066
BW	1	0.17450	0.174503	21.92	0.005
CW*CW	1	0.45855	0.458553	57.59	0.001
CS*CS	1	0.01886	0.018863	2.37	0.184
BW*BW	1	0.33083	0.330826	41.55	0.001
CW*CS	1	0.00337	0.003369	0.42	0.544
CW*BW	1	0.05699	0.056994	7.16	0.044
CS*BW	1	0.00916	0.009158	1.15	0.333
Error	5	0.03981	0.007962		
Lack-of-Fit	3	0.01217	0.004055	0.29	0.831
Pure Error	2	0.02765	0.013823		
Total	14	1.39086			
Model summary	S = 0.089	R^2^ = 97.14%	R^2^(adj) = 91.99%	R-sq(pred) = 81.53%

1 $$\begin{gathered} {\text{CR }}={\text{ }} - 0.00{\text{444}}0{\text{ }}+~0.00{\text{1731}}~{\text{CW }}+~0.0000{\text{48}}~{\text{CS }} \hfill \\ \quad +~0.000{\text{22}}0~{\text{BW }} - ~0.000{\text{316}}~{\text{CW}}*{\text{CW }} - ~0.000000~{\text{CS}}*{\text{CS}} \hfill \\ \quad - ~0.0000{\text{99}}~{\text{BW}}*{\text{BW }}+~0.000000~{\text{CW}}*{\text{CS }} \hfill \\ \quad +~0.0000{\text{39}}~{\text{CW}}*{\text{BW }}+~0.00000{\text{3}}~{\text{CS}}*{\text{BW}} \hfill \\ \end{gathered}$$

2 $$\begin{gathered} {\text{WA }}={\text{ }} - 0.{\text{885 }}+~0.{\text{5}}0{\text{27}}~{\text{CW }}+~0.00{\text{66}}0~{\text{CS }}+~0.{\text{4242}}~{\text{BW}} \hfill \\ \quad {\text{ }} - ~0.0{\text{881}}~{\text{CW}}*{\text{CW }} - ~0.0000{\text{13}}~{\text{CS}}*{\text{CS }} - ~0.0{\text{748}}~{\text{BW}}*{\text{BW }} \hfill \\ \quad +~0.000{\text{193}}~{\text{CW}}*{\text{CS }}+~0.0{\text{298}}~{\text{CW}}*{\text{BW }} - ~0.000{\text{319}}~{\text{CS}}*{\text{BW}} \hfill \\ \end{gathered}$$

**Fig. 7:**
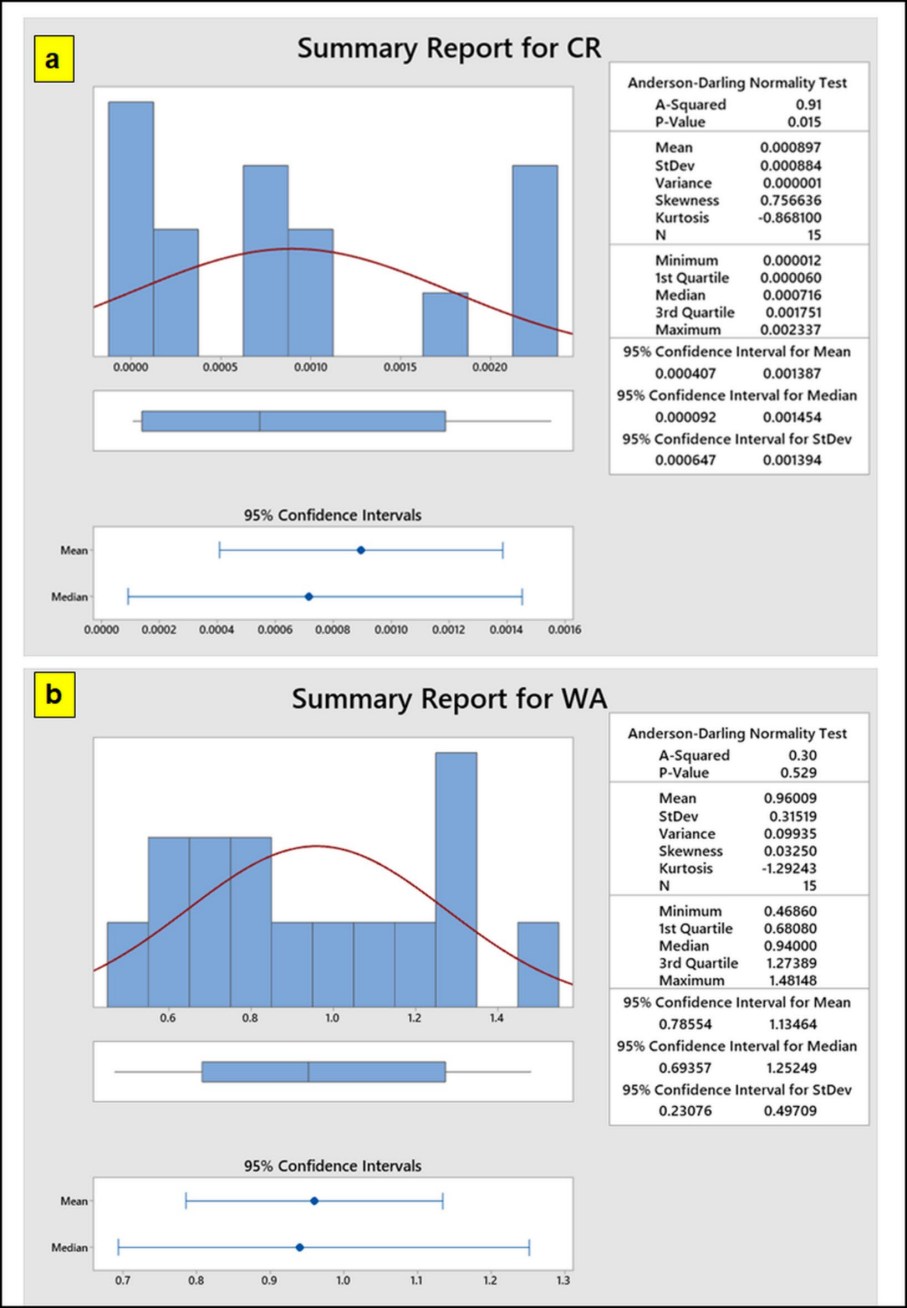
Histogram from mini plot of (a) CR (b) WA.

Figure 7 shows the histogram from mini plot of (a) CR (b) WA. The histogram, produced from the mini-plot for corrosion rates, is a helpful tool for visually assessing the performance of composite materials. It provides a thorough grasp of the influence of many elements on corrosion resistance by assisting in the identification of trends, variability and possible outliers. According to the histogram figure, the sample mean for CR 0.89 × 10^− 3^ and WA (0.96), is inside the 95% confidence interval^53,54^. The sample appears to provide strong evidence against the null hypothesis, as indicated by the modest p-value, which shows that the observed mean differs significantly from a predicted value.

**Fig. 8:**
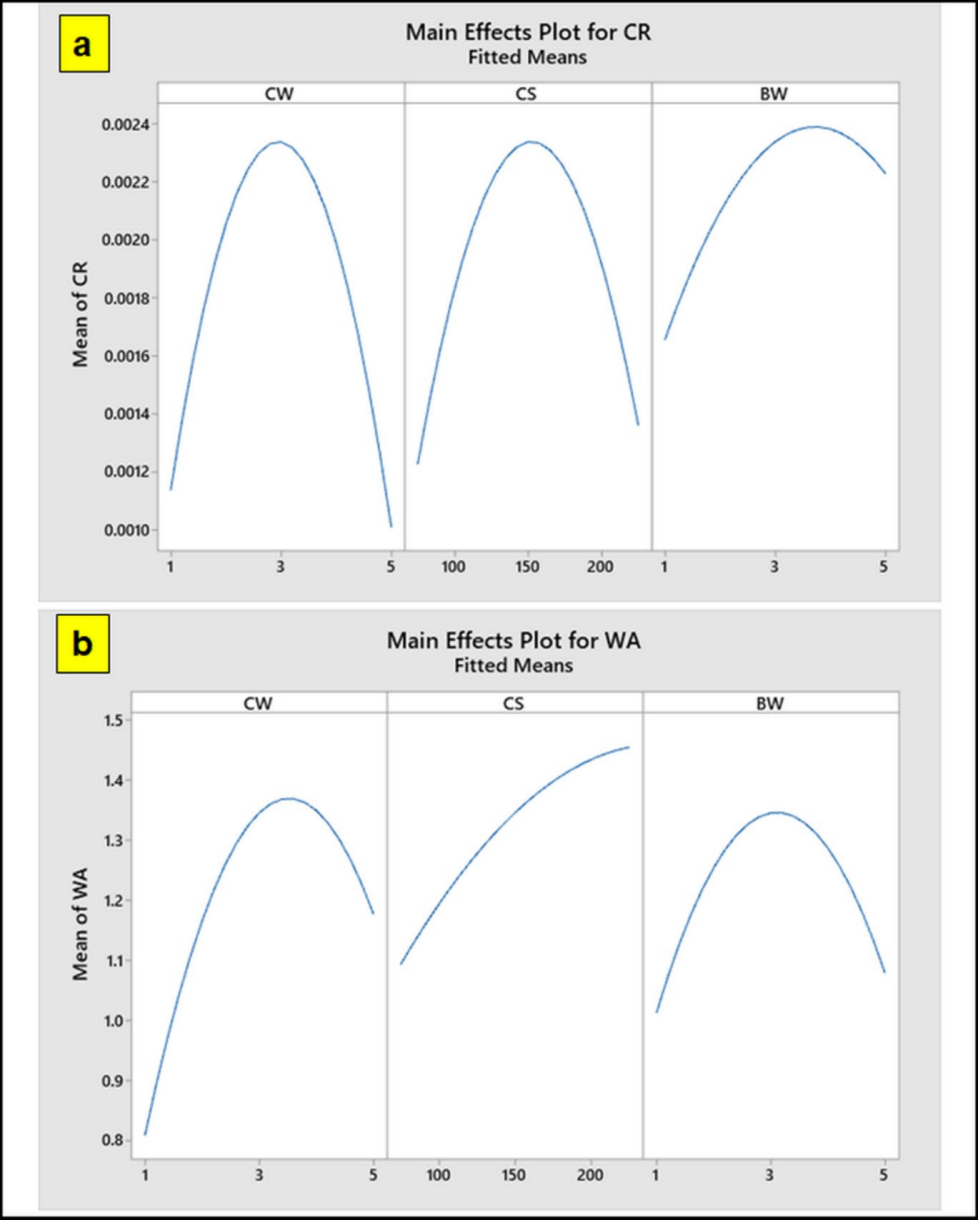
Mean effect plots for (a) CR (b) WA.

The main effect plot, shown in Fig. 8 (a) and (b), shows the various input parameters affecting the particulate polymer composites’ corrosion rate (CR) and water absorption (WA). The impact of the size of coir fillers, hexagonal boron nitride (hBN) and weight percentages of coir fiber on the material’s characteristics are particularly highlighted in this plot. According to the findings, adding coir and hBN fillers to the particulate polymer hybrid composites causes a rise in CR and WA. This suggests that the natural fillers are a factor in the material’s weakness to moisture and deterioration in harmful conditions. Furthermore, the particulate composites with the lowest CR and WA values have the least amount of coir fiber and hBN fillers in them. This implies that decreased filler content improves material performance, possibly as a result of improved porosity reduction and dispersion within the particulate polymer composite^42^. Plotting clearly demonstrates that adding 5 weight% of hBN causes the CR to drop, indicating that hBN functions as a powerful barrier against corrosive agents, improving the corrosion resistance of the composite. On the other hand, the plot shows that when the coir filler size rises from 1 to 5 weight%, the composite’s WA dramatically increases. The hydrophilic character of coir fibers, which promotes increased water absorption as the filler concentration rises, may be the cause of this increase in WA^55^. Additionally, the main effect plot shows that the mid-range levels of coir and hBN particle loading correspond to the maximum values of both CR and WA. This intermediate loading could result in a compromise between matrix integrity and filler interaction, which would lead to less-than-ideal performance for both water absorption and corrosion resistance.

**Fig. 9:**
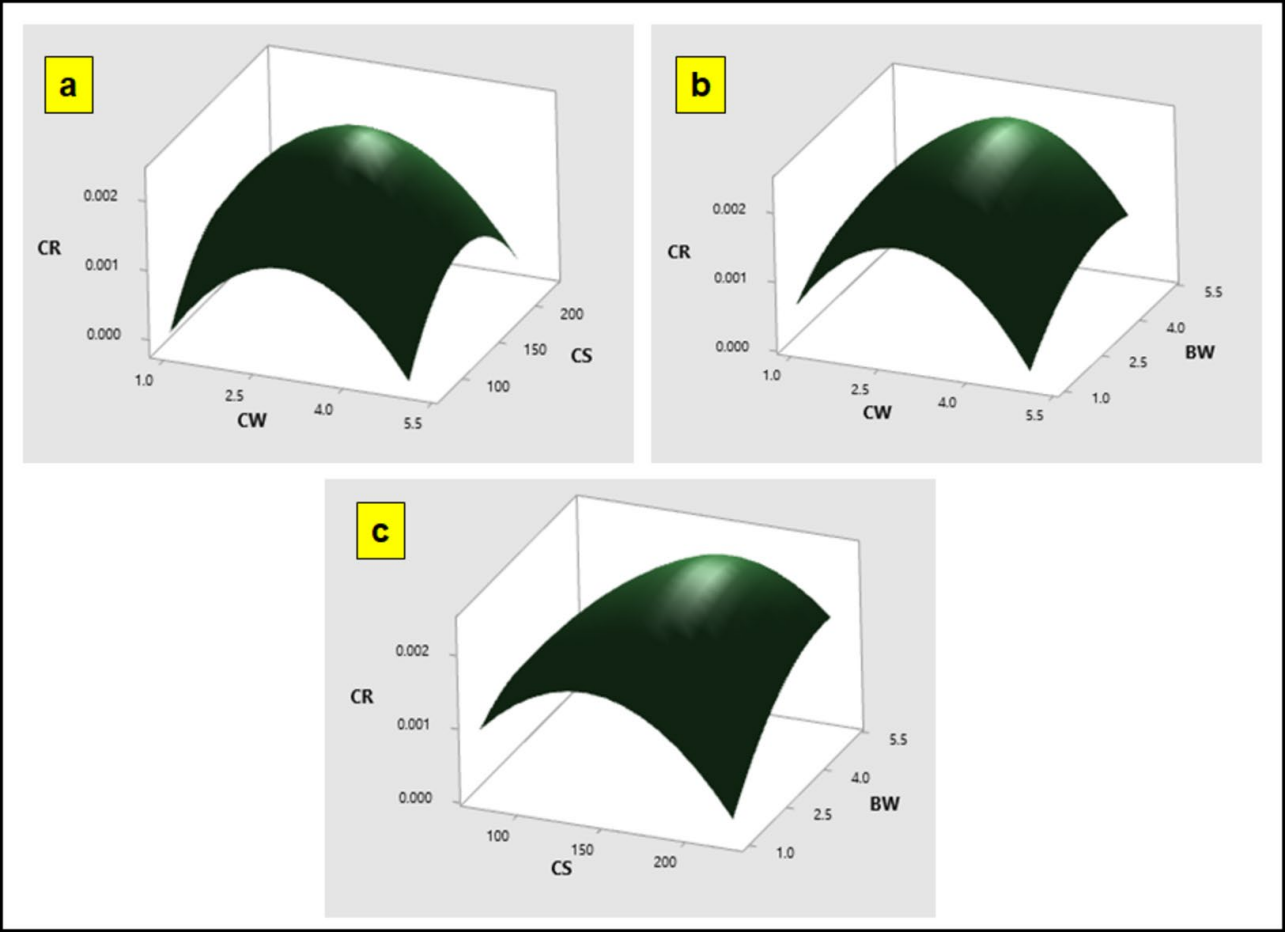
Response Optimization 3D plots of CR vs. (a)CS, CW (b)BW, CW (c) BW, CS.

**Fig. 10:**
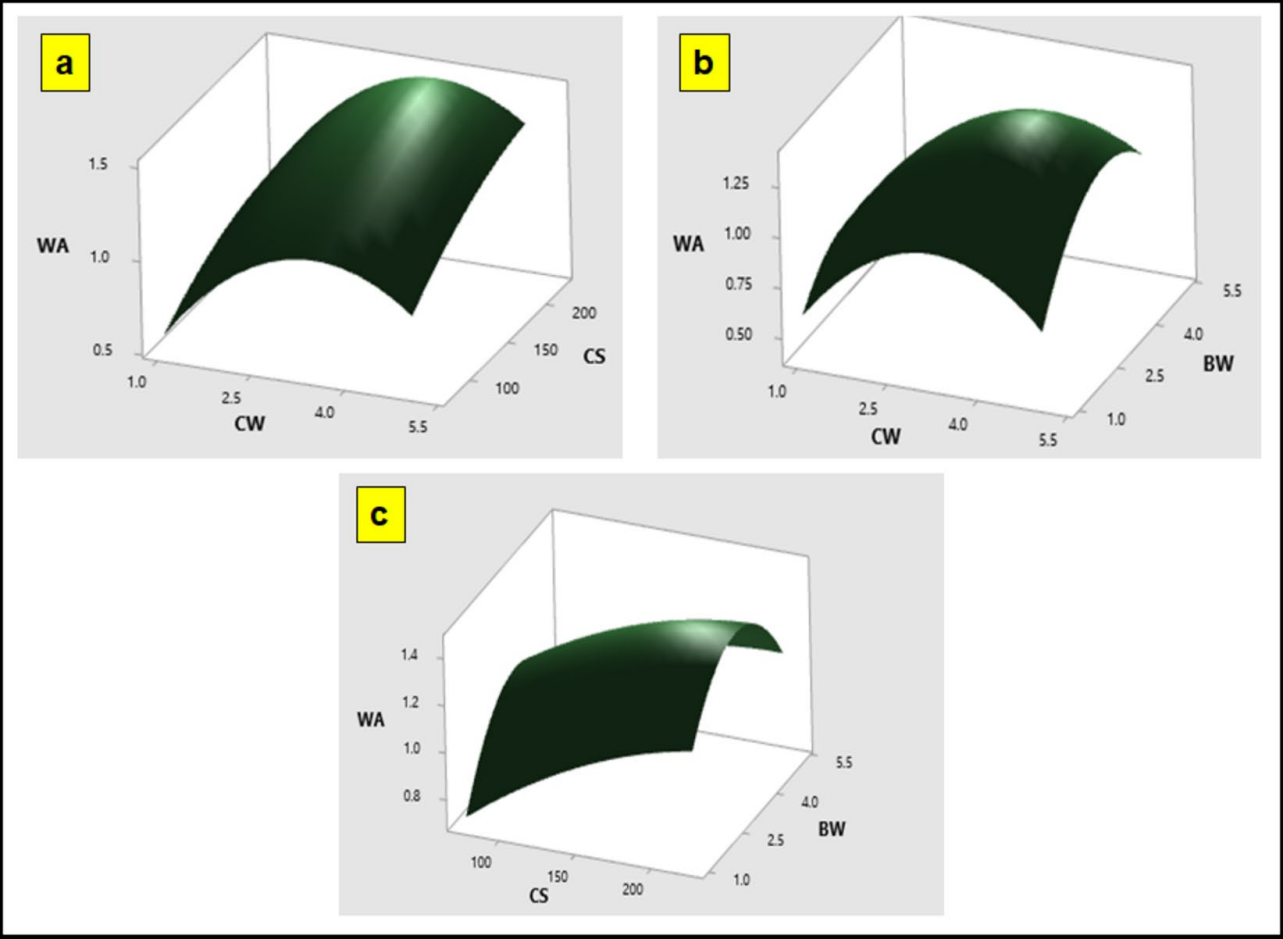
Response Optimization 3D plots of WA vs. (**a**) CS, CW (**b**) BW, CW (**c**) BW, CS.

To further understand the results, 3D response surface plots are utilized for the predicted model. These plots effectively demonstrate that the corrosion rate (CR) and water absorption (WA) are influenced by the chosen parameters. The 3D response surface graphs illustrate the interaction between two key factors affecting CR and WA: coir fiber size and hBN loading (Figs. 9 and 10). The plots clearly show that both CR and WA increase with higher coir fiber content. This is attributed to the high natural fiber content in the particulate polymer composites, which leads to poor wettability between the fiber and matrix. Additionally, as the concentration of coir particulate increased, the threshold value of coir size decreased, resulting in a rise in WA. This indicates that larger coir fiber sizes increase the composite’s capacity to absorb water. The study highlights that the optimal conditions for minimizing these effects are achieved with a coir fiber size of 75 μm and a coir content of 1 wt%.

#### Multiple response prediction

**Fig. 11:**
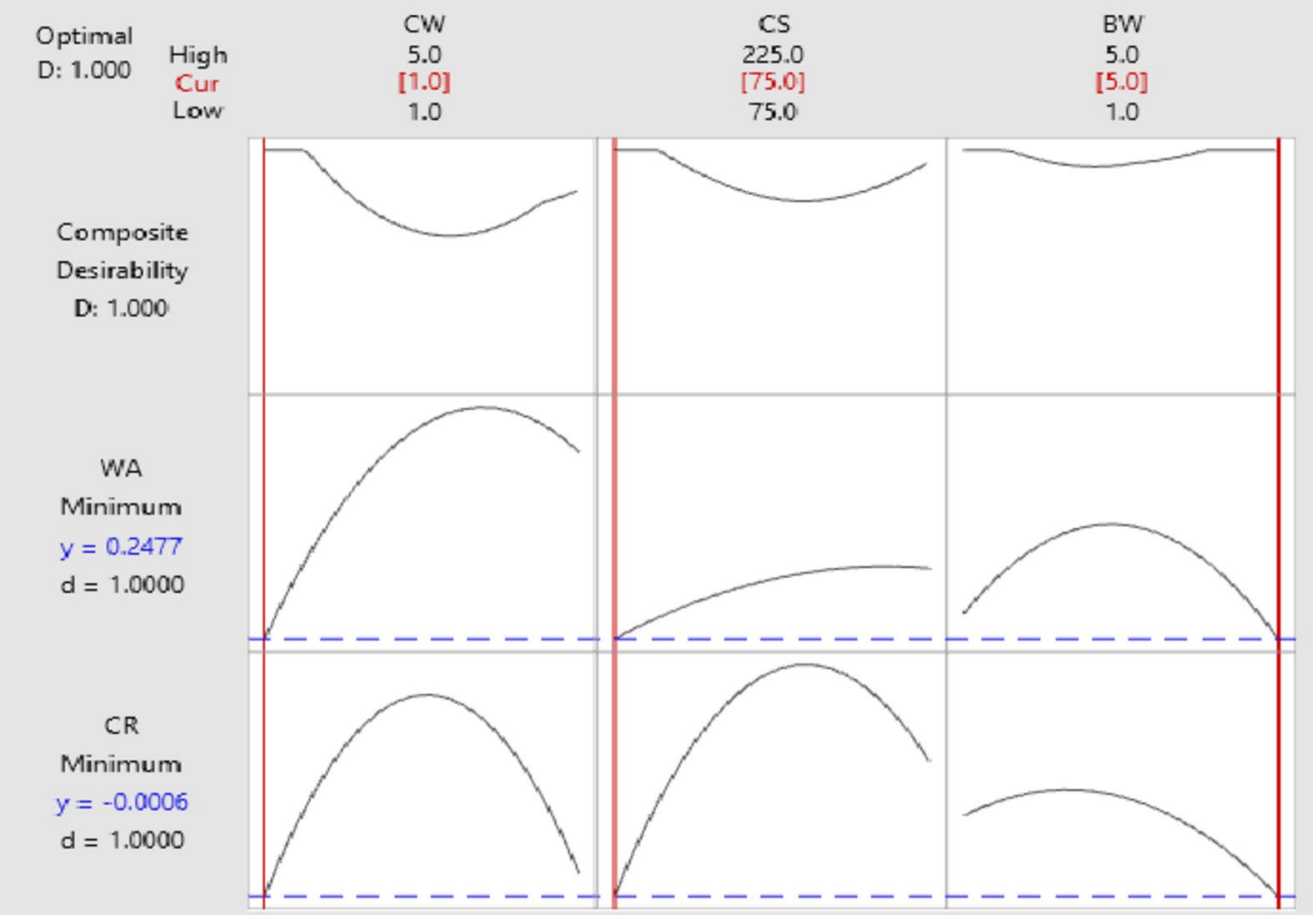
Response optimisation plot for corrosion rate and water absorption.

The multiple response prediction indicates a compromise between corrosion rate (CR) and water absorption (WA) for the specified variable values (CW = 1 wt%, CS = 75 μm and BW = 5 wt%) and results of Multiple Response Prediction Table 6. Figure 11 shows the response optimisation plot for corrosion rate and water absorption. The plot shows that decreasing the amount of coir slows down water absorption but partially increases corrosion rate. According to the optimisation plot, a reasonable balance can be achieved by utilising an intermediate percentage of hBN and a moderate amount of coir fiber to achieve acceptable levels of both CR and WA. This arrangement provides adequate resistance to corrosion while maintaining the composite’s ability to absorb moisture. The optimisation objectives for CR and WA are combined into a single score via the desirability index in the plot. When a value is closer to 1, it means that the chosen parameter settings are almost perfect for overall efficiency. The plot unequivocally shows that the best outcomes are achieved by carefully balancing the weight percentages of hBN and coir fiber, which minimises the rate of corrosion and water absorption.

**Table 6:** Results of multiple response prediction.

Response	Fit	SE Fit	95% CI	95% PI
WA	0.248	0.105	(-0.023, 0.519)	(-0.107, 0.603)
CR	-0.000622	0.000144	(-0.000992, -0.000252)	(-0.001107, -0.000137)

### Various metaheuristic algorithms optimum results

BBO, PSO, SSA and TLBO are the four metaheuristic algorithms used to find the best settings for reducing the rate of corrosion and water absorption in coir/hBN particulate polymer composite. The optimal strategy for obtaining the required material qualities is determined by comparing the ways in which each program investigates the solution space. To identify the ideal set of input parameters that provide the lowest CR and WA values, the method entails initialising, assessing and iteratively improving solutions.

#### Optimum process parameter using O_BBO

The Orthogonal Biogeography-Based Optimisation (O-BBO) approach is used to estimate the ideal process parameters for minimising corrosion rate (CR) and water absorption (WA) in the composite material. The results are shown in the Table 7. Using this optimisation method, the ideal set of input parameters is found to produce the required material qualities^39,43^. The table shows that coir fiber content, coir fiber size and hBN loading of 1%, 133.68 wt% and 4.85 wt% are the ideal parameter choices for minimising both CR (4.26 × 10^− 3^) and WA (0.44%). The lowest anticipated values for CR are obtained with these settings, indicating that this combination offers the optimum material property balance.

**Table 7:** Optimum process parameter using O_BBO.

*R*.No.	CW (wt%)	CS (µm)	BW (wt%)	CR (x10^− 3^) (mpy)	WA (%)
1	2.54	206.65	4.43	10.05	1.19
2	1.00	144.46	4.20	5.07	0.63
3	1.00	225.00	1.00	8.61	0.71
4	1.00	198.15	3.36	8.24	0.83
5	1.23	190.98	5.00	8.10	0.56
6	1.00	198.92	1.00	7.28	0.68
7	1.00	222.98	5.00	9.84	0.44
8	1.00	173.85	5.00	6.75	0.44
9	1.44	164.81	1.70	6.33	0.93
10	1.00	166.11	5.00	6.26	0.43
11	1.53	163.18	4.73	6.73	0.76
12	1.14	181.41	4.62	7.45	0.63
13	1.70	172.53	3.39	7.46	1.09
14	1.00	225.00	5.00	9.97	0.44
15	1.00	133.68	4.85	4.26	0.44
16	2.15	194.36	1.00	7.94	1.01
17	1.60	207.14	4.87	9.52	0.77
18	1.00	225.00	4.38	9.97	0.64
19	4.42	202.66	2.05	8.40	1.28
20	1.00	174.12	5.00	6.77	0.44
21	2.14	208.53	4.82	10.00	0.98
22	1.00	225.00	5.00	9.97	0.44
23	1.00	209.23	5.00	8.98	0.45
24	1.00	225.00	1.00	8.61	0.71
25	1.14	173.82	1.00	6.16	0.70
26	1.00	225.00	5.00	9.97	0.44
27	1.32	154.89	4.13	6.08	0.81
28	1.00	137.13	5.00	4.43	0.40
29	1.00	225.00	1.00	8.61	0.71
30	1.00	216.11	5.00	9.41	0.45
31	1.00	199.94	5.00	8.39	0.45

#### Particle swarm optimization (PSO) for CR and WA

A linear inertia weight variation is incorporated into Particle Swarm Optimisation (PSO) through the use of dynamic adaptation and MATLAB. The shortcomings of conventional regression models are addressed by this method, which finds it difficult to represent the complex and nonlinear behaviours that are frequently present in the investigations^40–42^. With each iteration, PSO a meta-heuristic evolutionary optimisation algorithm produces a population of viable solutions and gets closer to the global optimum (Table 8). PSO, quickly and with less computational effort, determines the global optimal parameter settings, in contrast to classical optimisation techniques that can become stuck in local optimal. PSO determines that a 1% coir fiber content, 133.68 μm coir fiber size and 4.85 wt% hBN loading are the ideal process parameters. The ideal values of 4.264 × 10^− 3^ mpy for corrosion rate (CR) and 0.44 for water absorption (WA) are obtained with these settings, which also minimise both of these parameters. The outcomes show that PSO is in identifying the ideal parameter combinations for enhanced material performance.

**Table 8:** Optimum process parameter using PSO.

*R*.No.	CW (wt%)	CS (µm)	BW (wt%)	CR (x10^− 3^) (mpy)	WA (%)
1	2.54	206.64	4.42	10.054	1.19
2	1	144.46	4.2	5.071	0.63
3	1	225	1	8.61	0.71
4	1	198.15	3.36	8.237	0.83
5	1.23	190.97	5	8.103	0.56
6	1	198.91	1	7.28	0.68
7	1	222.97	5	9.843	0.44
8	1	173.84	5	6.747	0.44
9	1.44	164.81	1.70	6.333	0.93
10	1	166.11	5	6.26	0.43
11	1.53	163.18	4.73	6.725	0.76
12	1.14	181.40	4.62	7.449	0.63
13	1.70	172.52	3.39	7.458	1.09
14	1	225	5	9.97	0.44
15	1	133.68	4.85	4.264	0.44
16	2.15	194.36	1	7.939	1.01
17	1.59	207.14	4.86	9.516	0.77
18	1	225	4.37	9.966	0.64
19	4.42	202.66	2.051	8.398	1.28
20	1	174.12	5	6.765	0.44
21	2.13	208.53	4.81	10.004	0.98
22	1	225	5	9.97	0.44
23	1	209.23	5	8.977	0.45
24	1	225	1	8.61	0.71
25	1.14	173.82	1	6.158	0.70
26	1	225	5	9.97	0.44
27	1.32	154.89	4.13	6.083	0.81
28	1	137.13	5	4.434	0.40
29	1	225	1	8.61	0.71
30	1	216.10	5	9.41	0.45
31	1	199.94	5	8.391	0.45

#### Optimum process parameter using SSO

The proposed method is compared in the experiments to the standard SSO over 31 benchmark functions. In terms of fitness function (mean best, worst and standard), SSO performs better than the other techniques. In order to achieve globally optimal solutions that meet the objective functions’ requirements, the SSO parameters are self-tuned during the optimisation process^41,56,57^. To achieve creatively ideal settings, the algorithms are run 31 times (Table 9). Deng’s statistical ranking approach is used to determine the suitable optimal parameter settings acquired from each run. Deng’s ranking method, particularly known as Grey Relational Analysis (GRA), is a technique derived from grey system theory. This method is primarily used to analyze and rank the relationships among various factors based on their similarity to a reference sequence. The process begins by defining a reference sequence (the dependent variable) and comparison sequences (independent variables). The next step involves calculating the grey relational coefficients, which quantify the degree of similarity between the reference and comparison sequences. These coefficients are derived using specific equations that consider the resolution coefficient, typically set at 0.5. Subsequently, the grey relational degrees are computed from these coefficients, reflecting the overall association strength of each factor with the reference. Finally, the factors are ranked based on their grey relational degrees; a higher degree indicates a stronger relationship, leading to a higher rank. This method has gained widespread application across various fields, including healthcare and risk assessment, due to its effectiveness in handling uncertainty and incomplete information in data analysis^58^.

SSO determines that a 1% coir fiber content, 225 μm coir fiber size and 5 wt% hBN loading are the ideal process parameters. The ideal values almost all of 9.97 × 10^− 3^ for corrosion rate (CR) and 0.44% for water absorption (WA) are obtained with these settings, which also minimise both of these parameters.

**Table 9:** Optimum process parameter using SSO.

*R*.No.	CW (wt%)	CS (µm)	BW (wt%)	CR (x10^− 3^) (mpy)	WA (%)
1	1	225	5	9.97	0.44
2	1	217.09	4.63	9.498	0.57
3	1	225	5	9.97	0.44
4	1	225	5	9.97	0.44
5	1	225	5	9.97	0.44
6	1	225	5	9.97	0.44
7	1	225	5	9.97	0.44
8	1	225	4.70	9.978	0.54
9	1	225	5	9.97	0.44
10	1	225	5	9.97	0.44
11	1	225	1	8.61	0.71
12	1	225	5	9.97	0.44
13	1	225	5	9.97	0.44
14	1	225	5	9.97	0.44
15	1	225	5	9.97	0.44
16	1	225	5	9.97	0.44
17	1	225	5	9.97	0.44
18	1	225	5	9.97	0.44
19	1	225	5	9.97	0.44
20	1	225	5	9.97	0.44
21	1	225	5	9.97	0.44
22	1	225	5	9.97	0.44
23	1	225	5	9.97	0.44
24	1	225	5	9.97	0.44
25	1	225	5	9.97	0.44
26	1	225	5	9.97	0.44
27	1	225	5	9.97	0.44
28	1	225	5	9.97	0.44
29	1	225	5	9.97	0.44
30	1	225	5	9.97	0.44
31	1	225	5	9.97	0.44

#### Optimum process parameter using TLO

The aim of the study is to determine the ideal process parameters for particulate polymer composites by investigating the Teaching-Learning Optimisation (TLO) method, which mimics the interactions between a teacher and students in a classroom^52,57^. First, certain response qualities like water absorption (WA) and corrosion resistance (CR) are optimised. Following the combination of these attributes, a composite objective function (Z) is created and TLO is used to optimise it. Based on the data, TLO performs better than Genetic Algorithm (GA). In basic TLO, students and teachers share knowledge, but it is expanded to incorporate student self-learning. A relatively recent addition to TLO, multi-objective TLO incorporates numerous professors and students for more efficient. Table 10 shows that coir fiber content, coir fiber size and hBN loading of 1%, 131.7 wt% and 5 wt% are the ideal parameter choices for minimising both CR (4.09 × 10^− 3^) and WA (0.39%). The lowest anticipated values for CR are obtained with these settings, indicating that this combination offers the optimum material property balance.

**Table 10:** Optimum process parameter using TLO.

*R*.No.	CW (wt%)	CS (µm)	BW (wt%)	CR (mpy)	WA (%)
1	1	225.00	5	9.97	0.44
2	1	154.90	5	5.55	0.42
3	1	205.33	5	8.73	0.45
4	1.50	225.00	4.81	10.55	0.74
5	1	162.31	4.72	6.08	0.52
6	1	214.58	5	9.31	0.45
7	1	225.00	5	9.97	0.44
8	1	180.25	1	6.33	0.65
9	1	225.00	1	8.61	0.71
10	1	131.75	5	4.10	0.39
11	1	225.00	5	9.97	0.44
12	1.63	186.60	3.97	8.29	1.00
13	1	188.77	5	7.69	0.45
14	1.14	196.34	4.96	8.35	0.53
15	1	219.95	3.81	9.60	0.77
16	1	202.81	1.39	7.72	0.77
17	1.03	216.89	4.21	9.50	0.70
18	1	225.00	5	9.97	0.44
19	1	225.00	1	8.61	0.71
20	1	215.21	5	9.35	0.45
21	2.50	216.83	5	10.69	1.02
22	1	194.93	4.87	8.09	0.49
23	1	198.46	1	7.26	0.68
24	2.11	225.00	5	11.02	0.91
25	1	225.00	5	9.97	0.44
26	1	225.00	5	9.97	0.44
27	1	225.00	5	9.97	0.44
28	1	220.87	1	8.40	0.70
29	1	170.87	5	6.56	0.44
30	1.55	216.38	5	10.05	0.71
31	1	225.00	5	9.97	0.44

**Fig. 12:**
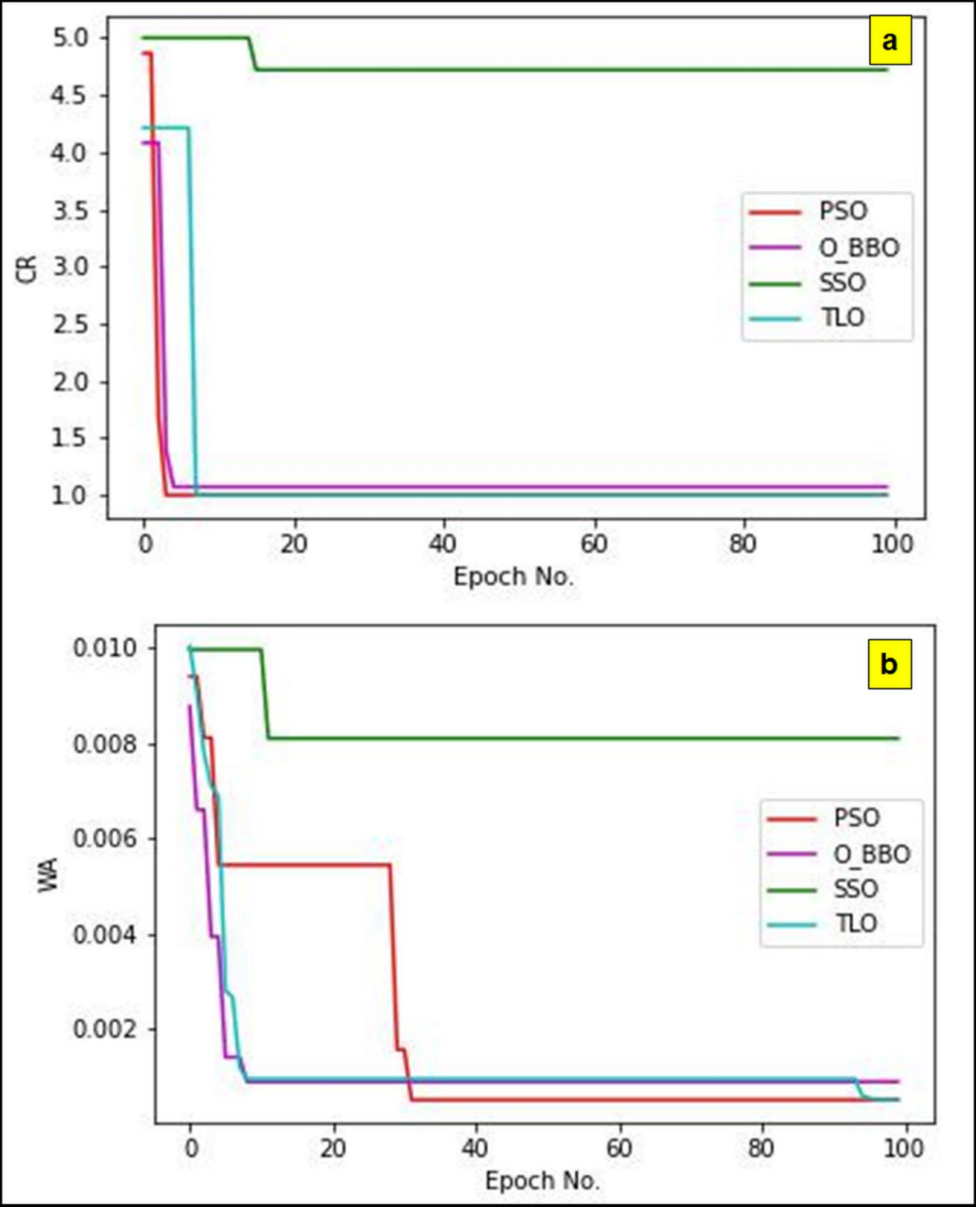
Convergence Plot for the response (a) CR (b)WA.

The suggested techniques are frequently used in the training phase to reduce the values of WA (water absorption) and CR (corrosion rate), which increase the meta-heuristic algorithms model’s prediction accuracy. Several optimisation techniques are run 100 times at once in order to find the best parameters for the meta-heuristic algorithms. Figure 12 (a, b) shows the convergence graphs correspond to the methods PSO (Particle Swarm Optimisation), O_BBO (Optimised Biogeography-Based Optimisation), SSO (Salp Swarm Optimisation) and TLO (Teaching-Learning Optimisation). The convergence rates in Fig. 12 are obtained by monitoring the best fitness value (corresponding to responses CR and WA) achieved by each algorithm across the 100 iterations. These plots illustrate the algorithms’ ability to minimize the objective function over time, providing insights into their convergence behaviour and efficiency. The maximum iteration count of 100 is selected based on preliminary testing and to ensure computational feasibility, especially for scenarios requiring multiple runs. However, 100 iterations may be a relatively small value, potentially limiting the algorithms’ ability to fully explore the solution space and demonstrate their efficiency^59^. The relationship between the number of iterations and meta-heuristic algorithms outputs, like CR and WA, is depicted in these graphs. Deng’s statistical ranking approach is used to determine the optimum configuration among the different optimal parameter values that are obtained from each experiment run. With this method, a numerical number known as Deng’s value is used to rank each solution according to well it performs in reaching the targeted results, such as minimising wear amount and corrosion rate (WA). The solutions are methodically graded based on their Deng’s values, as indicated in Table 11, creating an obvious hierarchy of efficacy. The Salp Swarm Optimisation (SSO) method performs better in this evaluation than PSO, O_BBO and TLO, the other three optimisation strategies. Among all the methods examined, the SSO approach produces the lowest CR and WA values in addition to converging more efficiently. This suggests that in order to optimise the meta-heuristic algorithms model parameters and get the highest overall performance, the SSO method is the most effective. It also shows that it is the best at minimising wear and corrosion. A higher Deng’s Value is generally preferred, as it reflects better performance^60^. Among the algorithms, SSO outperforms the others in minimizing both corrosion resistance (CR) and water absorption (WA), as evidenced by its superior Deng’s Value. The maximum Deng’s value obtained for SSO is 0.68 and all other Algorithms Perform closely. The most significant order of Algorithm Performance using Deng’s Method is SSO, O_BBO, PSO and TLO.

**Table 11:** Comparison of Algorithm Performance using Deng’s Method.

Algorithm	MoSo	CT	CR-CITN	WA-CITN	Deng’s Value
O_BBO	3.193	2	2	1.451	0.674
PSO	2.161	3	1.822	2.919	0.652
SSO	2.322	1	3.467	2.806	0.680
TLO	2.322	4	2.709	2.822	0.646
Probability	0.0052	4.97E-20	3.19E-07	4.66E-06	
Null hypothesis	Rejected	Rejected	Rejected	Rejected	
Significant/Not Significant	Significant	Significant	Significant	Significant	

For Deng’s Value, a higher value is generally better. SSO’s higher Deng’s Value suggests that it has effectively optimized the objectives, resulting in better overall performance in minimizing both CR (Corrosion Resistance) and WA (Water Absorption) compared to the other algorithms.

## Conclusions

1. This study examines the impact of particulate loading (1–5 wt%) and particle size (100–200 μm) of coir and hBN on corrosion rates and water absorption properties. Sample 14 shows the lowest corrosion rate (0.01 × 10⁻³ mpy), likely due to the optimal hBN and coir fiber proportions enhancing durability.
2. O_BBO, PSO, SSO and TLO algorithms are used to optimize the operating parameters of particulate polymer composites, aiming to improve corrosion resistance (CR) and water absorption (WA) properties. Moreover, MATLAB code is used to solve this multi-objective optimization problem.
3. It is found that hBN significantly improves the corrosion resistance (CR) and water absorption (WA) properties of the particulate polymer composite. The histogram shows that the sample mean for CR (0.89 × 10⁻³) and WA (0.96) lies within the 95% confidence interval.
4. The results of the confirmation studies demonstrate that the proposed optimization strategy can effectively predict optimal parameters, with the experimental values for CR and WA closely matching those predicted by the SSO algorithm. The ideal values of 9.97 × 10^− 3^ for corrosion rate (CR) and 0.44% for water absorption (WA) are obtained with these settings, which also minimise both these parameters.
5. The highest Deng’s value of 0.68 for SSO is attained and all other algorithms perform reasonably well. Using Deng’s Method, the most important order of algorithm performance is SSO, O_BBO, PSO and TLO.

## Data Availability

The datasets used and/or analysed during the current study available from the corresponding author on reasonable request.
